# Contemporary management of ventricular electrical storm in Europe: results of a European Heart Rhythm Association Survey

**DOI:** 10.1093/europace/euac151

**Published:** 2022-10-05

**Authors:** Enrico Baldi, Giulio Conte, Katja Zeppenfeld, Radosław Lenarczyk, Jose M Guerra, Michal M Farkowski, Carlo de Asmundis, Serge Boveda

**Affiliations:** Division of Cardiology, Fondazione IRCCS Policlinico San Matteo, Pavia, Italy; Cardiocentro Ticino Institute, Ente Ospedaliero Cantonale, Via Tesserete 48, Lugano 6900, Switzerland; Department of Cardiology, Heart Lung Centre, Leiden University Medical Centre, Leiden, The Netherlands; Division of Medical Sciences in Zabrze, Department of Cardiology, Congenital Heart Diseases and Electrotherapy, Silesian Center for Heart Diseases, The Medical University of Silesia, Zabrze, Poland; Department of Cardiology, Hospital de la Santa Creu i Sant Pau, IIB SANT PAU, CIBERCV, Universitat Autónoma de Barcelona, Barcelona, Spain; II Department of Heart Arrhythmia, National Institute of Cardiology, Warsaw, Poland; Heart Rhythm Management Centre, Postgraduate Program in Cardiac Electrophysiology and Pacing, Universitair Ziekenhuis Brussel – Vrije Universiteit Brussel, European Reference Networks Guard-Heart, Brussels, Belgium; Heart Rhythm Management Centre, Postgraduate Program in Cardiac Electrophysiology and Pacing, Universitair Ziekenhuis Brussel – Vrije Universiteit Brussel, European Reference Networks Guard-Heart, Brussels, Belgium; Cardiology-Heart Rhythm Management Department, Clinique Pasteur, Toulouse, France

**Keywords:** Electrical storm, Ventricular arrhythmias, Arrhythmic storm, Catheter ablation, Autonomic modulation, EHRA survey

## Abstract

Electrical storm (ES) is a predictor of mortality, and its treatment is challenging. Moreover, not all potential therapeutic strategies are available in all hospitals, and a standardized approach among European centres is lacking. The aim of this European Heart Rhythm Association (EHRA) survey was to assess the current management of patients with ES both in the acute and post-acute phases in 102 different European centres. A 20-item online questionnaire was sent out to the EHRA Research Network Centres. The median number of patients with ES treated annually per centre is 10 (IQR 5–15). The possibility of using autonomic modulation (e.g. percutaneous stellate ganglion block or thoracic epidural anaesthesia) for the acute ES treatment is available in only 29.3% of the centres. Moreover, although over 80% of centres perform ventricular tachycardia ablation, this procedure is available 24/7 in only 16.5% of the hospitals. There is a significant heterogeneity among centres regarding the availability of AADs and their use before deciding to proceed with a non-AAD strategy; specifically, 4.4% of centres use only one drug, 33.3% use two drugs, and 12.2% >two drugs, while about 50% of the centres decide based on individual patient’s characteristics. Regarding the type of AADs used for the acute and post-acute management of ES patients, important variability is reported depending upon the underlying heart disease. Most patients considered for percutaneous ablation have structural heart disease. Only 46% of centres refer patients to psychological counselling after ES.

**What’s new?**

- A standardized approach among European centres for treating electrical storm (ES) is lacking.
- Ventricular tachycardia ablation 24 h/day and 7 days/week is available in only 16.5% of the centres for the acute ES treatment and autonomic modulation (percutaneous stellate ganglion block or thoracic epidural anaesthesia) is available in only 39.2%.
- There is a significant heterogeneity among centres regarding the availability of AADs and their use before deciding to proceed with a non-AAD strategy.

## Introduction

Electrical storm (ES) is defined as the occurrence of three or more episodes of sustained ventricular arrhythmia [ventricular tachycardia (VT) or ventricular fibrillation (VF)] occurring within 24 h, requiring either anti-tachycardia pacing (ATP) or cardioversion/defibrillation, with each event separated by at least 5 min.^1–6^ Electrical storm is considered a significant predictor of mortality, irrespective of the underlying heart disease and the history of previous VT/VF.^7^

The management of ES is challenging, and a multidisciplinary and multi-faceted approach is often required to ensure an effective treatment.^8^ Anti-arrhythmic drug (AAD) therapy is the first-line choice for treating an ES, but is often ineffective, especially in the first hour from the arrhythmic onset.^9^ Therefore, in most cases other interventions are needed, both in the acute phase and after patient stabilization.^10^ Acute management includes implantable cardiac defibrillator (ICD) reprogramming,^4^ deep sedation, or general anaesthesia,^11,12^ autonomic modulation through thoracic epidural anaesthesia ^13^ or stellate ganglion block,^14–16^ extracorporeal membrane oxygenation (ECMO) for haemodynamically unstable patients,^17,18^ and urgent catheter ablation.^10,19^ Once the patient is stable, elective catheter ablation (endocardial and/or epicardial),^20,21^ optimization of AAD therapy, cardiac sympathetic denervation,^22^ and non-invasive cardiac ablation ^23–25^ may be helpful in preventing ES recurrences.

Some therapeutic strategies for ES may not be available in all hospitals. A standardized approach among European centres is lacking and it is unknown how ES is managed across centres. Furthermore, 2015 ESC guidelines on the management of ventricular arrhythmias do not provide any specific recommendations or structured approach for patients with ES.^26^

The aim of this European Heart Rhythm Association (EHRA) survey was to assess the current management of patients with ES in the acute and post-acute phases in different European centres. Moreover, the availability of different pharmacological and non-pharmacological interventions was investigated.

## Methods

The EHRA Scientific Initiatives Committee conducted the present centre-based survey. A 20-item online questionnaire (Supplementary material online, *File S1*) was developed to collect information about the current therapeutic management of patients with ES in Europe. The first draft of the questionnaire was prepared by E.B. and G.C. and was then reviewed by experts (K.Z., R.L., S.B.). The link was sent out to the EHRA Research Network Centres. A total of 102 respondents completed the questionnaire included in this analysis; hence the results are reported as number and percentage of 102, unless specified otherwise. In case of missing response to a specific question, the total number of replies collected for that question is provided in the respective figure legend.

The respondents represented centres from the 20 European countries: Austria, Belgium, Bulgaria, Croatia, France, Germany, Greece, Ireland, Israel, Italy, Malta, The Netherlands, Poland, Romania, Serbia, Spain, Sweden, Switzerland, Turkey, and UK. Among participating centres, 64.4% were university hospitals, 28.7% non-university public hospitals, and 6.9% private hospitals.

### Infrastructures for electrical storm management

The reported median number of patients with ES treated annually per centre was 10 (IQR 5–15). The possibility of performing acute percutaneous coronary intervention (PCI) 24/7 was reported by 87.2% of respondents, acute PCIs available only during daytime and workdays by 7.8%, and no availability of PCI in 5%. The vast majority of centres performed ICD implantation (95.1%).

Regarding the specific type of acute haemodynamic support, intra-aortic balloon pump (IABP) was available in 84.3%, percutaneous mechanical support (e.g. Impella) in 54.9%, ECMO in 64.7%, while 6.9% of the hospitals had no available haemodynamic support. A total of 79 respondents (77.4%) reported that >1 specialist are usually involved in the treatment of ES, while the remaining 22.6% stated that there is usually only one specialist to manage ES: electrophysiologists being present in 87.3% of cases, followed by general cardiologists (56.8%), Intensive Cardiac Care specialists (56.8%), anesthesiologists (28.4%), and cardiac surgeons (6.8%).

### Electrophysiology laboratory (Ep Lab)

The following are a list of EP procedures available in the different institutions: 84.3% of the centres performed complex endocardial ablations including VT ablation, 1% complex endocardial ablations but no VT ablation, 3.9% conventional ablation procedures (without 3D mapping), and 2.9% diagnostic procedures only (e.g. electrophysiological studies). Epicardial ablation was available in 66.3% of the centres who perform VT ablation, while surgical ablation in 30.2% of them. No EP procedures were performed in 7.8% of centres.

Among the 85 centres performing catheter-based acute management of electrical storm, ablation was available only during daytime and workdays in 83.5%, or 24/7 in the remaining 16.5%.

### Acute management of Electrical storm

Concerning the strategies available for the acute management of ES, device (ICD/CRT) programming was available in the vast majority of centres (94.1%), followed by deep sedation (83.3%), implant of temporary pacemaker to avoid bradycardia-induced ventricular arrhythmias (81.4%), general anaesthesia (74.5%), less-common used pharmacotherapy (e.g. mexiletine, isoproterenol) (74.5%) and acute catheter ablation (71.6%).

In contrast, autonomic modulation [e.g. percutaneous stellate ganglion block (PSGB) or thoracic epidural anaesthesia] was only available in a small proportion of centres (39.2%); among them, 82.5% used to perform PSGB and 25% thoracic epidural anaesthesia.

### Pharmacological strategy

**Figure 1:**
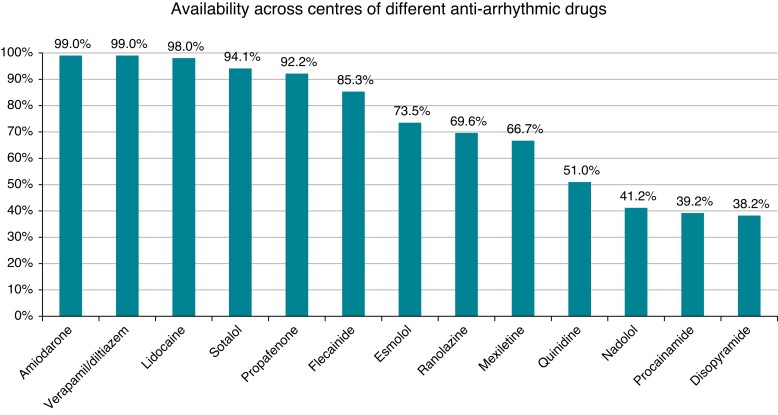
Anti-arrhythmic drugs available in the European countries.

A list of available anti-arrhythmic drugs (AADs) is presented in *Figure 1*. Amiodarone, propafenone, verapamil/diltiazem, lidocaine, and sotalol were available in more than 90% of the centres. The use of specific drugs in an individual patient according to the presumed underlying heart disease that caused the ES is presented in *Table 1*. The heterogeneity concerning the use of AADs is highlighted in the table.

**Table 1:** Drugs considered in the acute treatment of electrical storm according to the presumed underlying heart disease

	Amiodarone	Beta-blockers	Flecainide	Procainamide	Propafenone	Verapamil/diltiazem	Quinidine	Lidocaine	Mexiletine
Chronic coronary artery disease	94.5%	96.7%	4.4%	20.9%	5.5%	18.7%	8.8%	71.4%	57.1%
Cardiomyophaties (dilated, hypertrophic, AVC)	95.6%	93.4%	14.3%	19.8%	4.4%	7.7%	12.1%	50.6%	49.5%
Brugada syndrome/early repolarization	17.9%	39.3%	4.8%	8.3%	2.4%	6.0%	67.9%	13.1%	8.3%
Acquired LQTS	9.0%	87.2%	5.1%	1.3%	1.3%	2.6%	5.1%	20.5%	19.2%
Inherited LQTS	7.2%	89.2%	14.5%	2.4%	3.6%	3.6%	3.6%	18.1%	26.5%
PVCs-triggered VF	77.9%	86.1%	34.9%	10.5%	26.7%	25.6%	10.5%	45.4%	25.6%
Unknown aetiology	92.9%	89.4%	18.8%	10.6%	15.3%	17.7%	11.8%	44.7%	30.6%

AVC, arrhythmogenic ventricular cardiomyopathy; LQTS, long QT syndrome; PVC, premature ventricular contractions; VF, ventricular fibrillation.

The number of drugs administered before deciding to proceed with a non-AAD strategy varied between centres; 4.4% of centres reported using only one drug, 33.3% two drugs, and 12.2% >2 drugs. The remaining half of the centres answered that the number of drugs used depended on the patients’ characteristics (i.e. left ventricular ejection fraction, heart failure, haemodynamic status, chronic obstructive pulmonary disease).

### Non-pharmacological strategies

The different non-AAD therapy strategies in case of pharmacological treatment failure and according to the presumed underlying heart disease are presented in *Table 2*. Although catheter ablation was the most chosen strategy in patients with coronary artery disease or premature ventricular contractions (PVCs)-induced VF, deep sedation or general anaesthesia were, in general, the most consistently chosen therapies and the main ones in patients with Brugada syndrome or unknown aetiology. Temporary pacing was the most frequently used therapy for patients with LQTS.

**Table 2:** Preferred non-anti-arrhythmic drugs strategy in case of pharmacological treatment failure according to the presumed underlying heart disease, in the acute treatment of electrical storm

	Deep sedation	Intubation/general anesthesia	Percutaneous mechanical support (e.g. Impella)	Intra-aortic balloon pump	Extracorporeal membrane oxygenator (ECMO)	Autonomic modulation	Temporary PM	Acute catheter ablation
Chronic coronary artery disease	59.1%	58.0%	19.3%	25.0%	25.0%	18.2%	34.1%	77.3%
Cardiomyopathies (dilated, hypertrophic, AVC)	65.5%	60.9%	23.0%	17.2%	26.4%	26.4%	28.7%	60.9%
Brugada syndrome/early repolarization	66.7%	51.3%	5.1%	2.6%	9.0%	14.1%	23.1%	38.5%
Acquired LQTS	60.8%	53.2%	5.1%	3.8%	13.9%	21.5%	68.4%	8.9%
Inherited LQTS	64.6%	57.0%	3.8%	2.5%	12.7%	29.1%	62.0%	8.9%
PVCs-triggered VF	65.5%	52.4%	4.8%	2.4%	13.1%	20.2%	35.7%	70.2%
Unknown aetiology	72.8%	60.5%	7.4%	7.4%	21.0%	24.7%	25.9%	45.7%

AVC, arrhythmogenic ventricular cardiomyopathy; LQTS, long QT syndrome; PVC, premature ventricular contractions; VF, ventricular fibrillation; PM, pacemaker.

Deactivating appropriate anti-tachycardia ICD therapies was considered in all cases of ES by 18% of respondents, while 79.8% only considered it to avoid unnecessary therapies (e.g. repetitive, self-terminating VT), and 2.2% of respondents never considered deactivating anti-tachycardia therapies. Of those who considered deactivating ICD therapies, 48.1% deactivated ICD shock therapy only, whereas 43.2% deactivated both shock and ATP therapies. A total of 8.1% only used magnet placement to deactivate therapies.

### Post-acute and chronic management of ES

**Figure 2:**
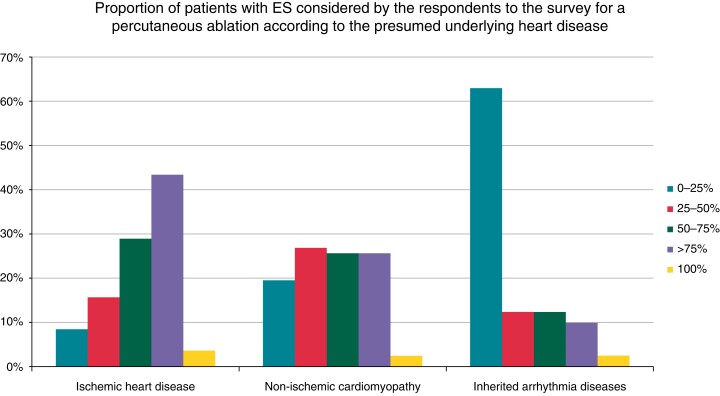
Proportion of patients with ES considered by the respondents to the survey for a percutaneous ablation, according to the presumed underlying heart disease.

Post-acute and chronic use of specific AADs to prevent ES recurrence according to the presumed underlying heart disease is presented in *Table 3*. A total of 27 centres (26%) reported having referred patients to other centres for the post-acute interventional management of ES (i.e. catheter ablation). Cardiac sympathetic denervation (thoracoscopic or surgical) was available in 20.6% of locations. The proportion of patients with a diagnosis of VT storm considered by the respondents for a percutaneous ablation, according to the underlying heart disease, is presented in *Figure 2*. Most patients considered for ventricular ablation were those with ischaemic heart disease, while those with an inherited arrhythmia condition were rarely referred for ablation. Of note, 47 centres (46%) declared referring patients to psychological counselling after an electrical storm.

**Table 3:** The use of specific anti-arrhythmic drugs, according to the presumed underlying heart disease in the post-acute and chronic management after arrhythmic storm

	Amiodarone	Cardio-selective beta-1-blockers	Flecainide	Sotalol	Procainamide	Propafenone	Verapamil	Procainamide	Ranolazine	Quinidine	Mexiletine	Disopyramide	Nadolol
Chronic coronary artery disease	93.8%	91.4%	2.5%	37.0%	3.7%	3.7%	7.4%	1.2%	24.7%	4.9%	35.8%	2.5%	8.6%
Cardiomyopathies (dilated, hypertrophic, AVC)	90.0%	87.5%	10.0%	37.5%	6.3%	2.5%	5.0%	0.0%	10.0%	8.8%	27.5%	5.0%	11.3%
Brugada syndrome/early repolarization	16.9%	39.4%	4.2%	1.4%	0.0%	2.8%	1.4%	1.4%	1.4%	71.8%	7.0%	0.0%	2.8%
Acquired LQTS	4.4%	86.8%	4.4%	4.4%	0.0%	1.5%	4.4%	0.0%	4.4%	1.5%	10.3%	0.0%	23.5%
Inherited LQTS	5.4%	81.1%	9.5%	1.4%	1.4%	0.0%	0.0%	0.0%	1.4%	5.4%	18.9%	2.7%	29.7%
PVCs-triggered VF	62.8%	87.2%	26.9%	25.6%	3.9%	23.1%	19.2%	2.6%	1.3%	15.4%	20.5%	0.0%	23.1%
Unknown aetiology	78.4%	86.5%	18.9%	20.3%	1.4%	9.5%	13.5%	1.4%	8.1%	8.1%	16.2%	0.0%	9.5%

## Discussion

The findings of this survey highlight several features of the current management of ES across Europe: (i) acute autonomic modulation is available in a relatively small proportion of centres; (ii) although more than 80% of centres perform VT ablation, availability of this therapy for 24 h/day and 7 days/week is reported in only a small percentage of centres; (iii) there is significant heterogeneity among centres about the use of AADs before referring patients for a non-AAD strategy; (iv) the type of AAD used for the acute and post-acute management of ES patients is variable and depends on the underlying heart disease; (v) psychological counselling is considered in nearly half of the patients who have experienced an ES; (vi) deep sedation and/or general anaesthesia is the most consistently chosen strategy throughout the spectrum of aetiologies.

Our survey confirms that ES is an important clinical problem in daily cardiology practice, as all centres report treating at least one patient every month. However, the condition could be overestimated since many of the respondents could be tertiary centres where these patients are referred to. Despite the fact the treatment of ES involves more than one specialist in about three-fourths of the centres, the role of electrophysiologists appears central, as they were involved in about 90% of cases.

Unfortunately, since many gaps in knowledge and evidence exist in this topic, standardizing treatment for ES patients is difficult.^26^ This is also confirmed in our survey, where a high heterogeneity was present among centres concerning the different therapeutic strategies to treat these patients.

### Autonomic modulation for the acute management of electrical storm

For the acute management of ES, autonomic modulation using PSGB or thoracic epidural anaesthesia is considered and performed only in one-third of the centres. However, the adrenergic nervous system’s hyperactivity plays a significant role in favouring ventricular arrhythmias ^27–29^ and the maintenance of the vicious circle, especially during recurrent ICD-shocks.^30^ The possibility of acutely reducing ventricular arrhythmias by blocking the left stellate ganglion has been known for decades.^31^ Recent reports from different centres have demonstrated the potential therapeutic effect of autonomic modulation in dramatically reducing arrhythmic recurrences during ES using thoracic epidural anaesthesia^13^ or PSGB^14,15,32,33^ in patients with different heart conditions (either ischaemic, non-ischaemic, and genetically determined cardiopathies) that caused the arrhythmic storm. This low rate of acute use of autonomic modulation has different possible explanations: among them, the scarce knowledge about the possibility of using such techniques for anti-arrhythmic purposes, the perception that catheter ablation is of easier applicability and the fact that electrophysiologists believe that special skills, often of formal anesthesiologic pertinence, are required to perform these techniques safely. Although thoracic epidural anaesthesia requires a trained anesthesiologist, PSGB can be safely performed by cardiologists using only anatomic landmarks without imaging guidance,^15,33^ as known since the Leriche and Fontain studies of the early 1900s.^34^ The reported rate of complications is very low, suggesting that this type of technique should become part of the modern electrophysiologists’ expertise in treating arrhythmic storm patients.

### Cather ablation in patients with electrical storm

Currently, urgent catheter ablation is a Class I-B recommendation for the treatment of patients presenting with arrhythmic storm, especially those with scar-related heart disease, left ventricular systolic dysfunction, or idiopathic VF triggered by PVC originating from the Purkinje system.^26^ Our survey highlights that only a minority of centres offers catheter ablation 24/7 despite the fact the majority perform this procedure. In the vast majority of centres, catheter ablation is available only during daytime and workdays; as a result, other strategies are needed to acutely stabilize the patients while waiting for an interventional strategy or an early referral to another centre where ablation is available in higher-risk patients.

### Anti-arrhythmic drug therapy

Our results highlight differences among centres regarding the availability of anti-arrhythmic drugs, especially those of less-common use, but that could be useful to treat an ES patient both acutely (e.g. procainamide, quinidine, and esmolol) or in the post-acute management (e.g. mexiletine and sotalol). Moreover, as expected, there is a significant variability in anti-arrhythmic drug use according to the presumed underlying heart disease, which shows the difficulty in standardizing the treatment of patients with ES. A special role is reserved for beta-blockers, which are used by ∼80–90% of centres for all types of heart disease (except Brugada syndrome).^35,36^

A great variability regarding the number of drugs commonly used in different centres before referring the patient to a non-AAD strategy is also shown in this study. Importantly, only a few centres used more than two AADs before using a non-AAD therapy, with most deciding according to the patient’s characteristics. These differences between centres probably reside in the availability and expertise in using non-AADs strategies for treating this type of patient and in the lack of clear and standardized guideline-based recommendations.

### Other acute and post-acute management aspects

Almost all the centres proceed to deactivate at least direct current-shock therapies. This seems to be very important in managing such patients as repeated ICD-shocks may enhance sympathetic activity, trigger additional arrhythmia,^30,37^ cause major anxiety, and even increase the risk of mortality.^38^

Haemodynamic mechanical support is available almost in all the centres, and this represents an option to be considered in the early management of haemodynamically unstable ES patients,^10^ also as a bridge to catheter ablation.^39^

Concerning the post-acute and chronic management of ES patients, a non-negligible percentage of centres refer their patients to another centre to perform VT ablation. These points out the importance of enhanced collaborations among centres to treat ES patients and ensure the best chance of survival, also in the acute setting. Ventricular tachycardia ablation is effective in reducing recurrences of arrhythmic storms and ventricular arrhythmias in patients with structural heart disease and in improving their prognosis.^20,21^ According to this survey, the percentage of patients referred to VT ablation is different according the underlying heart disease, with a greater percentage of patients referred to an interventional procedure among those with ischaemic heart disease, followed by those with non-ischemic cardiomyopathies. This is in line with current recommendations, which favour VT ablation in patients with a scar-related mechanism.^6,26^

Finally, nearly half of the centres refer patients to psychological counselling after electrical storm. This represents an important issue, often neglected by the clinicians, which should be further improved. It has been already highlighted, indeed, that ES patients are at higher risk of developing psychological disorders,^7^ in particular severe anxiety and depression.^40^ Therefore, based on published data, psychological counselling should be warranted to all ES patients after clinical stabilization.

## Conclusions

The treatment of ES represents a challenge, and the electrophysiologist plays a central role in managing these patients. A substantial heterogeneity is highlighted among European centres, especially concerning the availability and use of autonomic modulation techniques. Although it is difficult to standardize the treatment of such patients, further efforts are warranted to provide specific recommendations in future guidelines and to increase the expertise of European electrophysiologists in the use of non-anti-arrhythmic drugs strategies. Moreover, multicentric registries and efforts to better evaluate the role of autonomic modulation to control ES are needed.

## Supplementary material

Supplementary material is available at Europace online.

## Supplementary Material

euac151_Supplementary_Data

## References

[1] Credner SC , Klingenheben T, Mauss O, Sticherling C, Hohnloser SH. Electrical storm in patients with transvenous implantable cardioverter-defibrillators: incidence, management and prognostic implications. J Am Coll Cardiol 1998;32:1909–15. 9857871 10.1016/s0735-1097(98)00495-1

[2] Greene M , Newman D, Geist M, Paquette M, Heng D, Dorian P. Is electrical storm in ICD patients the sign of a dying heart? Outcome of patients with clusters of ventricular tachyarrhythmias. Europace 2000;2:263–9. 11227599 10.1053/eupc.2000.0104

[3] Exner DV , Pinski SL, Wyse DG, Renfroe EG, Follmann D, Gold M et al Electrical storm presages nonsudden death: the antiarrhythmics versus implantable defibrillators (AVID) trial. Circulation 2001;103:2066–71. 11319196 10.1161/01.cir.103.16.2066

[4] Hohnloser SH , Al-Khalidi HR, Pratt CM, Brum JM, Tatla DS, Tchou P et al Electrical storm in patients with an implantable defibrillator: incidence, features, and preventive therapy: insights from a randomized trial. Eur Heart J 2006;27:3027–32. 17050586 10.1093/eurheartj/ehl276

[5] Haegeli LM , Bella PD, Brunckhorst CB. Management of a patient with electrical storm: role of epicardial catheter ablation. Circulation 2016;133:672–6. 26884622 10.1161/CIRCULATIONAHA.115.016336

[6] Cronin EM , Bogun FM, Maury P, Peichl P, Chen M, Namboodiri N et al 2019 HRS/EHRA/APHRS/LAHRS expert consensus statement on catheter ablation of ventricular arrhythmias. Europace 2019;21:1143–4. 31075787 10.1093/europace/euz132 PMC7967791

[7] Guerra F , Shkoza M, Scappini L, Flori M, Capucci A. Role of electrical storm as a mortality and morbidity risk factor and its clinical predictors: a meta-analysis. Europace 2014;16:347–53. 24096960 10.1093/europace/eut304

[8] Muser D , Santangeli P, Liang JJ. Management of ventricular tachycardia storm in patients with structural heart disease. World J Cardiol 2017;9:521. 28706587 10.4330/wjc.v9.i6.521 PMC5491469

[9] Levine JH , Massumi A, Scheinman MM, Winkle RA, Platia EV, Chilson DA et al Intravenous amiodarone for recurrent sustained hypotensive ventricular tachyarrhythmias. J Am Coll Cardiol 1996;27:67–75. 8522712 10.1016/0735-1097(95)00427-0

[10] Kowlgi GN , Cha YM. Management of ventricular electrical storm: a contemporary appraisal. Europace 2020;22:1768–80. 32984880 10.1093/europace/euaa232

[11] Liu Q , Kong AL, Chen R, Qian C, Liu SW, Sun BG et al Propofol and arrhythmias: two sides of the coin. In: (ed.), Acta pharmacologica Sinica. 2011. p817–23. 10.1038/aps.2011.42 PMC3505762 21642950

[12] Burjorjee JE , Milne B. Propofol for electrical storm; a case report of cardioversion and suppression of ventricular tachycardia by propofol. Can J Anesth 2002;49:973–7. 12419728 10.1007/BF03016886

[13] Do DH , Bradfield J, Ajijola OA, Vaseghi M, Le J, Rahman S et al Thoracic epidural anesthesia can be effective for the short-term management of ventricular tachycardia storm. J Am Heart Assoc 2017;6:e007080. 29079570 10.1161/JAHA.117.007080 PMC5721785

[14] Fudim M , Qadri YJ, Waldron NH, Boortz-Marx RL, Ganesh A, Patel CB et al Stellate ganglion blockade for the treatment of refractory ventricular arrhythmias. JACC Clin Electrophysiol 2020;6:562–71. 32439042 10.1016/j.jacep.2019.12.017

[15] Savastano S , Dusi V, Baldi E, Rordorf R, Sanzo A, Camporotondo R et al Anatomical-based percutaneous left stellate ganglion block in patients with drug-refractory electrical storm and structural heart disease: a single-centre case series. Europace 2021;23:581–6. 33190159 10.1093/europace/euaa319

[16] Savastano S , Pugliese L, Baldi E, Dusi V, Tavazzi G, De Ferrari GM. Percutaneous continuous left stellate ganglion block as an effective bridge to bilateral cardiac sympathetic denervation. Europace 2020;22:606. 32034906 10.1093/europace/euaa007

[17] Baudry G , Sonneville R, Waintraub X, Lebreton G, Deguillard C, Mertens E et al Extracorporeal membrane oxygenation to support life-threatening drug-refractory electrical storm. Crit Care Med 2020:E856–63. 32796185 10.1097/CCM.0000000000004490

[18] Savastano S , Baldi E, Camporotondo R, Belliato M, Marinoni B, De Ferrari GM. Percutaneous stellate ganglion block and extracorporeal cardiopulmonary resuscitation: an effective and safe combination for refractory ventricular fibrillation. Europace 2020;22:148. 31257420 10.1093/europace/euz180

[19] Hayashi M , Miyauchi Y, Murata H, Takahashi K, Tsuboi I, Uetake S et al Urgent catheter ablation for sustained ventricular tachyarrhythmias in patients with acute heart failure decompensation. Europace 2014;16:92–100. 23858022 10.1093/europace/eut207

[20] Carbucicchio C , Santamaria M, Trevisi N, Maccabelli G, Giraldi F, Fassini G et al Catheter ablation for the treatment of electrical storm in patients with implantable cardioverter-defibrillators : short-and long-term outcomes in a prospective single-center study. Circulation 2008;117:462–9. 18172038 10.1161/CIRCULATIONAHA.106.686534

[21] Vergara P , Tung R, Vaseghi M, Brombin C, Frankel D, Di Biase L et al Successful ventricular tachycardia ablation in patients with electrical storm reduces recurrences and improves survival. Hear Rhythm 2018;15:48–55. 10.1016/j.hrthm.2017.08.022 28843418

[22] Dusi V , Gornbein J, Do DH, Sorg JM, Khakpour H, Krokhaleva Y et al Arrhythmic risk profile and outcomes of patients undergoing cardiac sympathetic denervation for recurrent monomorphic ventricular tachycardia after ablation. J Am Heart Assoc 2021;10:1–14. 10.1161/JAHA.120.018371 PMC7955320 33441022

[23] Cuculich PS , Schill MR, Kashani R, Mutic S, Lang A, Cooper D et al Noninvasive cardiac radiation for ablation of ventricular tachycardia. N Engl J Med 2017;377:2325–36. 29236642 10.1056/NEJMoa1613773 PMC5764179

[24] Dusi V , Vitolo V, Frigerio L, Totaro R, Valentini A, Barcellini A et al First-in-man case of non-invasive proton radiotherapy for the treatment of refractory ventricular tachycardia in advanced heart failure. Eur J Heart Fail 2021;23:195–6. 33179329 10.1002/ejhf.2056

[25] Robinson CG , Samson PP, Moore KMS, Hugo GD, Knutson N, Mutic S et al Phase I/II trial of electrophysiology-guided noninvasive cardiac radioablation for ventricular tachycardia. Circulation 2019;139:313–21. 30586734 10.1161/CIRCULATIONAHA.118.038261 PMC6331281

[26] Priori SG , Blomström-Lundqvist C, Mazzanti A, Bloma N, Borggrefe M, Camm J et al 2015 ESC guidelines for the management of patients with ventricular arrhythmias and the prevention of sudden cardiac death: the task force for the management of patients with ventricular arrhythmias and the prevention of sudden cardiac death of the Europe. Europace 2015;17:1601–87. 26318695 10.1093/europace/euv319

[27] Ajijola OA , Lux RL, Khahera A, Kwon O, Aliotta E, Ennis DB et al Sympathetic modulation of electrical activation in normal and infracted myocardium: implications for arrhythmogenesis. Am J Physiol – Hear Circ Physiol 2017;312:H608–21. 10.1152/ajpheart.00575.2016 PMC5402014 28087519

[28] Priori SG , Mantica M, Schwartz PJ. Delayed afterdepolarizations elicited in vivo by left stellate ganglion stimulation. Circulation 1988;78:178–85. 3383403 10.1161/01.cir.78.1.178

[29] Zipes DP , Barber MJ, Takahashi N, Gilmour RF. Influence of the autonomic nervous system on the genesis of cardiac arrhythmias. Pacing Clin Electrophysiol 1983;6:1210–20. 6195641 10.1111/j.1540-8159.1983.tb04459.x

[30] Tsuji Y , Heijman J, Nattel S, Dobrev D. Electrical storm: recent pathophysiological insights and therapeutic consequences. Basic Res Cardiol 2013;108:336. 23429935 10.1007/s00395-013-0336-2

[31] Schwartz PJ , Stone HL, Brown AM. Effects of unilateral stellate ganglion blockade on the arrhythmias associated with coronary occlusion. Am Heart J 1976;92:589–99. 983934 10.1016/s0002-8703(76)80078-6

[32] Tian Y , Wittwer ED, Kapa S, McLeod CJ, Xiao P, Noseworthy PA et al Effective use of percutaneous stellate ganglion blockade in patients with electrical storm. Circ Arrhythmia Electrophysiol 2019;12:e007118. 10.1161/CIRCEP.118.007118 31514529

[33] Meng L , Tseng CH, Shivkumar K, Ajijola O. Efficacy of stellate ganglion blockade in managing electrical storm: a systematic review. JACC Clin Electrophysiol 2017;3:942–9. 29270467 10.1016/j.jacep.2017.06.006 PMC5734652

[34] Leriche R , Fontaine R. L’Anesthesie isolee du ganglion etoile. Sa technique, ses indications, ses resultats. Press Medicale 1934;42:849–50.

[35] Chatzidou S , Kontogiannis C, Tsilimigras DI, Georgiopoulos G, Kosmopoulos M, Papadopoulou E et al Propranolol versus metoprolol for treatment of electrical storm in patients with implantable cardioverter-defibrillator. J Am Coll Cardiol 2018;71:1897–906. 29699616 10.1016/j.jacc.2018.02.056

[36] Farkowski MM , Karlinski M, Pytkowski M et al Mexiletine for recurrent ventricular tachycardia in adult patients with structural heart disease and implantable cardioverter defibrillator: an EHRA systematic review. Europace 2022;24:1504–11. 35851797 10.1093/europace/euac101

[37] Elsokkari I , Tsuji Y, Sapp JL, Nattel S. Recent insights into mechanisms and clinical approaches to electrical storm. Can J Cardiol 2022;38:439–53. 34979281 10.1016/j.cjca.2021.12.015

[38] Poole JE , Johnson GW, Hellkamp AS, Anderson J, Callans DJ, Raitt MH et al Prognostic importance of defibrillator shocks in patients with heart failure. N Engl J Med 2008;359:1009–17. 18768944 10.1056/NEJMoa071098 PMC2922510

[39] Mathuria N , Wu G, Rojas-Delgado F, Shuraih M, Razavi M, Civitello A et al Outcomes of pre-emptive and rescue use of percutaneous left ventricular assist device in patients with structural heart disease undergoing catheter ablation of ventricular tachycardia. J Interv Card Electrophysiol 2017;48:27–34. 27497847 10.1007/s10840-016-0168-8

[40] Sears SF , Hauf JD, Kirian K, Hazelton G, Conti JB. Posttraumatic stress and the implantable cardioverter-defibrillator patient what the electrophysiologist needs to know. Circ Arrhythmia Electrophysiol 2011;4:242–50. 10.1161/CIRCEP.110.957670 21505176

