# Supplementary material for: Contemporary management of ventricular electrical storm in Europe: results of a European Heart Rhythm Association Survey
Source: Europace. 2022 Oct 5;25(4):1277–83. doi: 10.1093/europace/euac151 (PMC10105853; doi:10.1093/europace/euac151)
Supplement: euac151_Supplementary_Data [file euac151_supplementary_data.pdf]

Introduction

Dear colleague,

The management of ventricular electrical storm is challenging and requires a multi-faceted approach to ensure a proper and effective treatment. To date, there is no uniform approach to electrical storm across Europe.
The aim of this EHRA survey is to assess how patients with electrical storm are treated both in the acute and post-acute phase, and to investigate the availability of different pharmacological and non-pharmacological strategies and adherence to current guidelines.

The survey will take approximately 5 minutes. Your answers will be anonymous.

Thank you in advance for your contribution!

* 1. GDPR Disclaimer

Your participation is anonymous.
We will not disclose your identity to any third party.

We comply with the European General Data Protection Regulation (GDPR) 2016/679. Any personal data processed in connection with this survey will be treated confidentially and only used by the ESC for the purposes of market research and not for promotion. Survey results will be kept for a maximum of 48 months for analysis and quality control purposes. We take all reasonable care to prevent any unauthorised access to your personal data. We respect your privacy and your right to access, modify, or remove your personal data. At any time, you can ask to know what personal data is being held. If you have any questions about data protection or require further information, please contact our data protection officer (DPO) at dpo@escardio.org.

You have the right to end your participation in this survey at any time.

Please confirm that you have read the above and agree to participate in this survey.

- ◯ yes
- ◯ no

2. In which country is your centre based?

3. Primary working environment

- University Hospital
- Non-University Hospital
- Private Hospital
- Private practice
- Other (please specify)

4. Is your institution a PCI centre?

- No
- Yes, only during daytime and workdays
- Yes, 24 hours a day, 7 days a week

5. Is your institution an ICD implanting centre?

- No
- Yes

6. Who is usually involved at your institution in the management of electrical storm? (Multiple answer possible)

- General Cardiologist
- Intensive Cardiac Care specialist
- Electrophysiologist
- Anaesthesiologist
- Cardiac surgeon
- Other (please specify)

7. What type of strategy is available at your institution for the acute management of electrical storm? (Multiple answer possible)

- Deep sedation
- General anaesthesia
- Mechanical hemodynamic support
- Autonomic modulation (percutaneous stellate ganglion block or thoracic epidural anaesthesia)
- Device (ICD/CRT) programming
- Temporary pacemaker implantation
- Acute catheter ablation
- Non-standard pharmacotherapy (mexiletine, isoproterenol)
- Other (please specify)

8. Which type of hemodynamic supports are available at your institution? (Multiple answer possible)

- [ ] Intra-aortic balloon pump (IABP)
- [ ] Left Ventricular Assist Device (LVAD)
- [ ] Percutaneous mechanical support (e.g. Impella)
- [ ] Extracorporeal membrane oxygenator (ECMO)
- [ ] None of the above
- [ ] I don’t know

9. Which type of autonomic modulation therapy is used in your centre for acute treatment of the electrical storm? (Multiple answer possible)

- [ ] Percutaneous stellate ganglion block
- [ ] Thoracic epidural anaesthesia
- [ ] Cardiac sympathetic denervation (thoracoscopic or surgical)
- [ ] No autonomic modulation therapy is performed at my centre

10. Which type of Electrophysiology procedures are available in your institution? (Multiple answer possible)

- [ ] Only diagnostic procedures (i.e. EP study)
- [ ] Conventional ablation procedures (non-3D mapping)
- [ ] Complex endocardial ablations (not including VT ablation)
- [ ] Complex endocardial ablations including VT ablation
- [ ] Epicardial ablations
- [ ] Surgical ablations
- [ ] None of the above

11. Are catheter ablation procedures available for the acute management of the electrical storm?

- ○ No
- ○ Yes, only during daytime and workdays
- ○ Yes, 24 hours a day, 7 days a week

12. Which drugs are available in your country? (Please select all drugs available)

- [ ] Amiodarone
- [ ] Flecainide
- [ ] Procainamide
- [ ] Propafenone
- [ ] Verapamil/diltiazem
- [ ] Quinidine
- [ ] Lidocaine
- [ ] Mexiletine
- [ ] Sotalol
- [ ] Ranolazine
- [ ] Disopyramide
- [ ] Nadolol
- [ ] Esmolol

ACUTE MANAGEMENT

13. Which drugs would you consider in the acute treatment of electrical storm in these categories of patients? Please read one line at a time and select all drugs that you may be using

	Amiodarone	Beta-blockers	Flecainide	Procainamide	Propafenone	Verapamil/diltiazem	Quinidine	Lidocaine	Mexiletine
Chronic coronary artery disease	☐	☐	☐	☐	☐	☐	☐	☐	☐
Cardiomyopathies (dilated, hypertrophic, AVC)	☐	☐	☐	☐	☐	☐	☐	☐	☐
Brugada syndrome / Early repolarization	☐	☐	☐	☐	☐	☐	☐	☐	☐
Acquired LQTS	☐	☐	☐	☐	☐	☐	☐	☐	☐
Inherited LQTS	☐	☐	☐	☐	☐	☐	☐	☐	☐
PVCs-triggered VF	☐	☐	☐	☐	☐	☐	☐	☐	☐
Unknown etiology	☐	☐	☐	☐	☐	☐	☐	☐	☐

14. How many drugs are commonly tested in your centre before deciding to proceed with a non-AAD strategy to acutely manage an electrical storm?

- ○ 1
- ○ 2
- ○ > 2
- ○ It depends on patient’s characteristics (i.e. EF, HF, hemodynamic status, COPD)

15. Which is your preferred strategy in case of pharmacological treatment failure? Please read one line at a time and select all procedures that you may be using

	Deep sedation	Intubation/ General anesthesia	Percutaneous mechanical support (e.g. Impella)	Intra-aortic balloon pump	Left ventricular assist device	Extracorporeal membrane oxygenator (ECMO)	Autonomic modulation	Temporary PM	Acute catheter ablation
Chronic coronary artery disease	☐	☐	☐	☐	☐	☐	☐	☐	☐
Cardiomyopathies (dilated, hypertrophic, AVC)	☐	☐	☐	☐	☐	☐	☐	☐	☐
Brugada syndrome / Early repolarization	☐	☐	☐	☐	☐	☐	☐	☐	☐
Acquired LQTS	☐	☐	☐	☐	☐	☐	☐	☐	☐
Inherited LQTS	☐	☐	☐	☐	☐	☐	☐	☐	☐
PVCs-triggered VF	☐	☐	☐	☐	☐	☐	☐	☐	☐
Unknown etiology	☐	☐	☐	☐	☐	☐	☐	☐	☐

16. Do you usually consider deactivating appropriate anti-tachycardia therapies in the acute management of electrical storm in patients with implantable cardioverter-defibrillators?

- ○ Always
- ○ Only to avoid unnecessary therapies (e.g. repetitive self-termination VT)
- ○ Never

17. How would you deactivate tachycardia-therapies in the acute management of electrical storm in patients with implantable cardioverter-defibrillators?

- ◯ Deactivation of ICD shock therapy only
- ◯ Deactivation of ICD shock therapy and anti-tachycardia pacing (ATP)
- ◯ Magnet placement (i.e. no programmer/PM specialist)
- ◯ I would not deactivate ICD

POST-ACUTE AND CHRONIC MANAGEMENT

18. Which drugs would you consider for post-acute and chronic management of the following patients? Please read one line at a time and select all drugs that you may be usin

	Amiodarone	Cardio-selective Beta-1-blockers	Flecainide	Sotalol	Procainamide	Propafenone	Verapamil	Procainamide	Ranolazine	Quinidine	Mexiletine	Disopyramide	Nadolol
Chronic coronary artery disease	☐	☐	☐	☐	☐	☐	☐	☐	☐	☐	☐	☐	☐
Cardiomyopathies (dilated, hypertrophic, AVC)	☐	☐	☐	☐	☐	☐	☐	☐	☐	☐	☐	☐	☐
Brugada syndrome / Early repolarization	☐	☐	☐	☐	☐	☐	☐	☐	☐	☐	☐	☐	☐
Acquired LQTS	☐	☐	☐	☐	☐	☐	☐	☐	☐	☐	☐	☐	☐
Inherited LQTS	☐	☐	☐	☐	☐	☐	☐	☐	☐	☐	☐	☐	☐
PVCs-triggered VF	☐	☐	☐	☐	☐	☐	☐	☐	☐	☐	☐	☐	☐
Unknown etiology	☐	☐	☐	☐	☐	☐	☐	☐	☐	☐	☐	☐	☐

19. How many patients with electrical storm do you manage annually?

0 — 20 or more

20. How many patients do you refer to other centres for the post-acute interventional management of electrical storm (i.e. catheter ablation)?

None or less than 5% — 100%

21. What proportion of patients with a diagnosis of VT storm is considered for a percutaneous ablation in your centre (including transfer to other centre for ablation)?

	0-25%	25-50%	50-75%	>75%	
Ischemic cardiomyopathy	○	○	○	○	○
Non-ischemic cardiomyopathy	○	○	○	○	○
Inherited arrhythmia diseases	○	○	○	○	○

22. How many patients do you refer to a psychological counselling after electrical storm?

None or less than 5% — 100

Thank you

Dear colleague,

Thank you very much for completing the survey.

Your input is very much appreciated and will provide us with invaluable insights.

Please click on “Done” to submit your responses.
